# Is there a pathogenic association between Fabry’s disease and IgA nephropathy? 

**DOI:** 10.5414/CNCS107994

**Published:** 2013-12-17

**Authors:** Shuichiro Fujinaga, Hitohiko Murakami, Mitsuru Kubota, Hiroshi Mochizuki, Toshiaki Shimizu

**Affiliations:** 1 Division of Nephrology, Saitama Children’s Medical Center,; 2 Division of Pathology, Saitama Children’s Medical Center,; 3 Division of General Pediatrics, Saitama Children’s Medical Center,; 4 Division of Metabolism and Endocrinology, Saitama Children’s Medical Center, Saitama-city Saitama, and; 5 Department of Pediatrics, Juntendo University School of Medicine, Tokyo, Japan

**Keywords:** Fabry’s disease, IgA nephropathy

## Abstract

Several cases of concurrent Fabry’s disease and IgA nephropathy have been reported, but the pathogenic association between these two diseases remains unclear. This is a report on the case of a girl with severe IgA nephropathy who was subsequently diagnosed with subclinical Fabry’s disease. An 11-year-old girl was admitted to our hospital with massive proteinuria and hematuria detected by urinary screening. An initial renal biopsy revealed severe IgA nephropathy with diffuse mesangial proliferation. She was treated with intravenous methylprednisolone pulses followed by 2 years of oral steroids. The treatment improved both the urinary abnormalities and the second renal biopsy findings. At the age of 15 years, mild proteinuria prompted us to perform a third renal biopsy, and histology revealed minor glomerular abnormalities. In addition, numerous myelin-like bodies were detected in podocytes by electron microscopy. The histological findings combined with the low level of α-galactosidase A activity led to the diagnosis of concomitant Fabry’s disease with IgA nephropathy. Our experience suggests that the initial massive proteinuria was not due to Fabry’s disease, but was rather a manifestation of coincidental IgA nephropathy. We speculate that the coexistence of IgA nephropathy and Fabry’s disease occurred by chance.

## Introduction 

Fabry’s disease is an X-linked disorder of glycosphingolipid catabolism resulting from deficient activity of the lysosomal enzyme α-galactosidase A. This defect leads to the accumulation of its substrates, mainly globotriaosylceramide (GL3), in the lysosomes of cells of different tissues. Although serious complications of the disease include renal manifestations such as proteinuria and renal failure, these are clinically silent during childhood in most female patients [1]. 

IgA nephropathy, defined as mesangial proliferative glomerulonephritis with mesangial predominant IgA deposits, is the most common primary glomerular disease worldwide. Previous reports have indicated that immunogenic GL3 accumulation in Fabry’s disease may contribute to the pathogenic role of IgA nephropathy, but the coexistence of the two diseases is very rare [2, 3, 4, 5, 6]. Here we report the case of a girl with severe IgA nephropathy who was diagnosed with Fabry’s disease 4 years later. 

## Case report 

**Figure 1.**
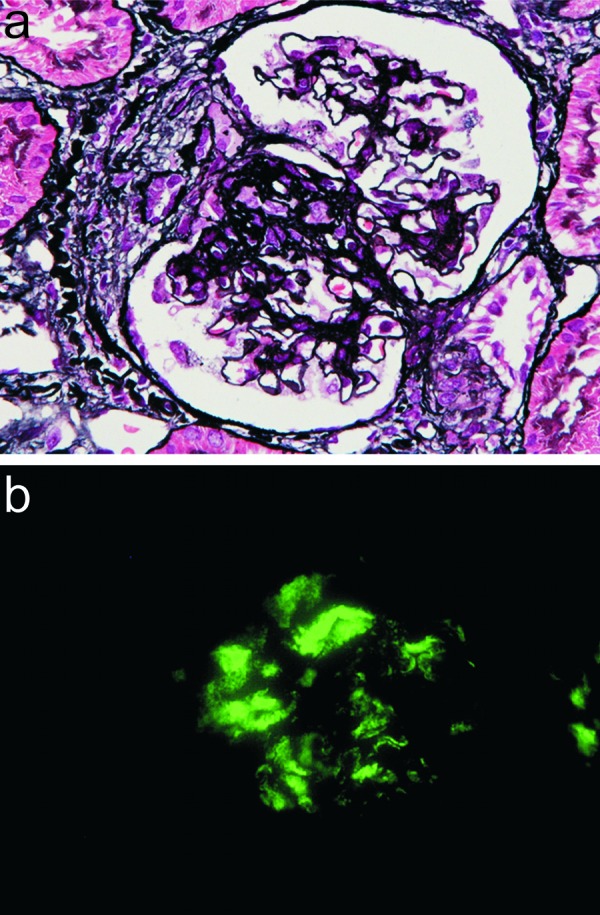
a: Light microscopy images from the first renal biopsy specimen showing mesangial proliferation with fibrocellular crescent formation. b: Immunofluorescence microscopy images showing coarse granular deposits of immunoglobulin A (3+).

An 11-year-old girl was admitted to our hospital with microscopic hematuria and nephrotic-range proteinuria detected by school urinary screening. The patient did not have any characteristic features of Fabry’s disease such as angiokeratoma or neuralgia. On admission, her physical examination was unremarkable (body weight 47.4 kg, body height 157.3 cm), and her blood pressure normal (106/60 mmHg). Laboratory investigations revealed hypoproteinemia (serum total protein 5.6 g/dL, serum albumin 2.9 g/dL, serum IgG 318 mg/dL). All other biochemical investigations were within normal limits: blood urea nitrogen 8 mg/dL, serum creatinine 0.43 mg/dL, IgA 111 mg/dL, C3 140 mg/dL (normal 84 – 151 mg/dL), C4 26 mg/dL (normal 17 – 40 mg/dL). Anti-hepatitis B surface antigen, anti-hepatitis C antibody, and anti-nuclear antibody were negative. Urinalysis findings were 3+ for occult blood and 3+ for the dipstick protein reaction; her 24-hour urine protein excretion was 2.5 g. Light microscopy analysis of the initial renal biopsy specimen revealed diffuse mesangial proliferative glomerulonephritis with fibrocellular crescents in 4 of 13 glomeruli (Figure 1a). Immunofluorescence revealed granular deposits of IgA (3+) and C3 (1+) in the mesangial areas (Figure 1b), and electron microscopy revealed paramesangial electron-dense deposits. Based on these findings, a diagnosis of severe IgA nephropathy was made. The patient was treated intravenously with three courses of methylprednisolone (20 mg/kg/day) pulse therapy (MPT), followed by 2 years of alternate-day prednisolone (initially 1 mg/kg and then tapered gradually) combined with an angiotensin receptor blocker (telmisartan 1 mg/kg/day). In addition, a tonsillectomy was performed 3 months after MPT. However, microscopic hematuria and mild proteinuria persisted. 

At the age of 13 years, the patient was re-admitted for a second renal biopsy to assess the therapeutic effect. All laboratory investigations were within normal limits: serum total protein 6.3 g/dL, serum albumin 4.0 g/dL, blood urea nitrogen 12 mg/dL, serum creatinine 0.44 mg/dL, IgA 108 mg/dL, C3 96 mg/dL, C4 26 mg/dL. Urinalysis revealed a 2+ value for occult blood and a dipstick protein reaction of 2+; the 24-hour urine protein excretion was 0.5 g. Light microscopy analysis of the second renal biopsy specimen revealed focal segmental mesangial proliferative glomerulonephritis. Immunofluorescence revealed granular deposits of IgA (2+) and C3 (1+) in the mesangial areas, and electron microscopy revealed paramesangial electron-dense deposits. After the second renal biopsy, the patient was additionally treated with three courses of MPT, and her microscopic hematuria disappeared within 6 months. 

**Figure 2.**
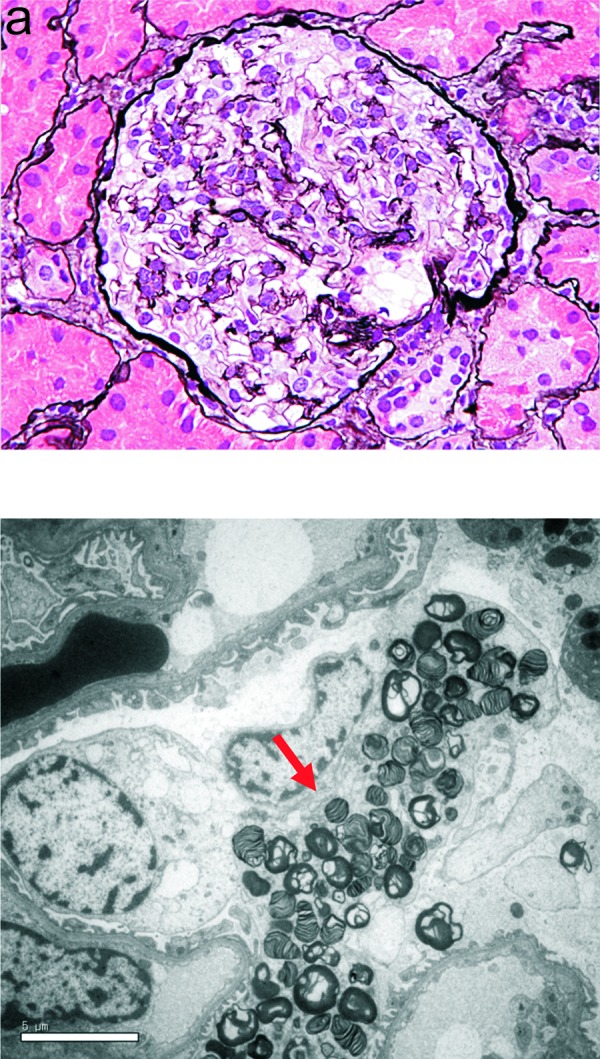
a: Light microscopy images from the third renal biopsy specimen showing minor glomerular abnormalities. b: Electron microscopy images from the third renal biopsy specimen showing numerous myelin-like bodies in podocytes (arrow).

At the age of 15 years, she was admitted for a third renal biopsy. On admission, her physical examination was unremarkable (body weight 55.9 kg, body height 165 cm), and her blood pressure normal (102/70 mmHg). Laboratory investigations were all within normal limits: serum total protein 6.6 g/dL, serum albumin 4.2 g/dL, blood urea nitrogen 11 mg/dL, serum creatinine 0.48 mg/dL, IgA 135 mg/dL, C3 121 mg/dL, C4 25 mg/dL. Urinalysis was negative for occult blood and the dipstick protein reaction was 2+; her 24-hour urine protein excretion was 0.7 g. Light microscopy analysis of the third renal biopsy specimen revealed minor glomerular abnormalities (Figure 2a). Immunofluorescence revealed granular deposits of IgA (2+) in the mesangial areas, and electron microscopy revealed paramesangial electron-dense deposits. In addition, numerous myelin-like bodies were first observed in podocytes (Figure 2b). Low serum α-galactosidase A activity (13.8 Agal U, cut-off value < 20) and increased GL3 excretion in the urine (0.54 mg/mgCr, control 0.1 – 0.4) were also observed. On the basis of these findings, a final diagnosis of concomitant IgA nephropathy (healing stage) and subclinical Fabry’s disease was made. 

## Discussion 

Here, we report the serial renal biopsy findings and clinical course of IgA nephropathy in a girl with Fabry’s disease. A number of cases of Fabry’s disease coexistent with immune disorders such as lupus nephritis, rheumatoid arthritis, and IgA nephropathy have been described in the literature [2, 3, 4, 5, 6, 7, 8]. The reason for the concomitant occurrence of autoimmune disorders and Fabry’s disease remains unclear. It has been suggested that immunogenic GL3 accumulation in patients with Fabry’s disease acts as a long-term antigenic stimulus that induces an autoimmune reaction [9, 10]. In addition, since GL3 is involved with lymphocyte function, one could imagine that deranged metabolism from alpha-galactosidase deficiency in Fabry’s disease could cause a shift in the balance in IgA glycosylation and lead to IgA nephropathy. 

Recently, Najafian et al. [11] suggested that Fabry’s disease and IgA nephropathy may be related because the concurrence of the two diseases appears to be more frequent than random. However, to date, only 5 cases of coexistent IgA nephropathy and Fabry’s disease have been reported in the literature [2, 3, 4, 5, 6]. Whybra et al. [2] described two adolescent sisters who were heterozygous for Fabry’s disease and had severe IgA nephropathy. Although the younger sister continued receiving enzyme replacement therapy (ERT) for Fabry’s disease from the age of 12 years, severe IgA nephropathy developed at the age of 16.5 years, indicating that early initiation of ERT does not necessarily prevent the development of IgA nephropathy. In our patient, treatment with MPT and tonsillectomy for severe IgA nephropathy resulted in a remarkable improvement in both urinary abnormalities and histological findings, suggesting that the initial nephrotic-range proteinuria was not due to Fabry’s disease, but was rather a manifestation of severe IgA nephropathy. Unexpectedly, numerous myelin-like bodies were first detected in podocytes by electron microscopy analysis of her third renal biopsy specimen, which led to the diagnosis of Fabry’s disease. Similarly, Kawamura et al. [4] reported a 28-year-old male patient with both IgA nephropathy and subclinical Fabry’s disease who did not show any clinical manifestations apart from abnormal urinalysis findings. Immunosuppressive treatment for severe IgA nephropathy ameliorated the histological and urinary abnormalities, but serial biopsies revealed GL3 accumulation. Furthermore, Yoshida et al. [5] reported the case of a 36-year-old female patient with massive proteinuria (2.5 g/day) associated with IgA nephropathy and subclinical Fabry’s disease. The patient did not have any characteristic features of Fabry’s disease such as angiokeratoma, neuralgia, or hypohidrosis. Recently, Shimohata et al. [3] reported on a 22-year-old male with classical Fabry’s disease and relatively mild IgA nephropathy. Although he experienced hydrohidrosis and neuralgia with fever and markedly low serum α-galactosidase levels, no severe mesangial proliferation or crescent formation was observed during light microscopy. From these case reports, including that of our patient, it appears that there is no clinicopathological correlation between IgA nephropathy and Fabry’s disease. This can be explained in terms of the different affected areas between the two diseases: electron microscopic abnormalities are mainly observed in the mesangial areas in IgA nephropathy, whereas podocytes are most prominently involved in Fabry’s disease. 

In conclusion, it appears that the concurrent IgA nephropathy and Fabry’s disease in our patient occurred by chance. However, immunosuppressive treatment for concomitant IgA nephropathy ameliorated the histological and urinary abnormalities, which may improve the renal prognosis of the patient. 

## Conflict of interest 

None declared.
